# Balancing Shading and Resistive Losses: 33% Efficient Perovskite/Silicon Tandem Solar Cells via Front‐Electrode Engineering

**DOI:** 10.1002/advs.78191

**Published:** 2026-10-06

**Authors:** Yuhui Ji, Fei Wang, Junjun Li, Yunren Luo, Haichuan Zhang, Qi Liu, Pengxu Chen, Kexin Yao, Qiang Shi, Fanying Meng, Liping Zhang, Chen Yang, Jiakai Liu, Zhengxin Liu, Wenzhu Liu, Jian Yu

**Affiliations:** ^1^ School of New Energy and Materials Southwest Petroleum University Chengdu China; ^2^ Shanghai Institute of Microsystem and Information Technology Chinese Academy of Sciences Shanghai China; ^3^ University of Chinese Academy of Sciences (UCAS) Beijing China; ^4^ Department of Mathematics Shanghai Normal University Shanghai China; ^5^ School of Chemistry and Chemical Engineering Institute of Advanced Inter‐Disciplinary Studies Chongqing University Chongqing China; ^6^ Xinjiang Technical Institute of Physics and Chemistry Chinese Academy of Sciences Urumqi China

**Keywords:** carrier extraction, carrier losses, metal electrodes, perovskite/silicon tandem solar cell, transparent conductive oxide (TCO)

## Abstract

Perovskite/silicon tandem solar cells (PSTSCs) have broken the efficiency ceiling of single‐junction solar cells, standing as a core technology for next‐generation photovoltaics. Nevertheless, carrier losses at the front electrode, composed of transparent conductive films and metal grids, are often underestimated and hinder further performance improvement. Herein, we synergistically optimized the transparent electrode and front metal grid to reduce interfacial carrier loss while balancing optical shading and series resistance losses. Tungsten‐doped indium oxide (IWO) with a lower work function and suitable energy alignment was adopted to replace conventional indium zinc oxide (IZO), improving carrier extraction and the device's fill factor (FF). Using our self‐developed simulation tool, we quantified grid‐induced power loss and systematically investigated how grid width and spacing affect photovoltaic performance. Guided by simulations, tandem cells with varied grid geometries were fabricated and tested. The optimized device delivered a champion power conversion efficiency (PCE) of 33.06% (certified 32.28%), with an FF of 80.87%, a short‐circuit current density (*J_SC_*) of 20.72 mA/cm^2,^ and an open‐circuit voltage (*V_OC_*) of 1.973 V. This work mitigates the understudied front‐electrode carrier losses in PSTSCs and offers a feasible route toward scalable high‐performance tandem photovoltaics.

## Graphical abstract

Front‐electrode co‐optimization combining tungsten‐doped indium oxide (IWO) transparent electrode and simulation‐guided metal grid geometry boosts perovskite/silicon tandem solar cell performance. The low work function IWO improves interfacial carrier extraction, while tailored grid parameters balance optical shading and electrical resistive losses. This combined strategy delivers a champion power conversion efficiency of 33.06 %, establishing practical design guidelines for high‐efficiency tandem photovoltaics.

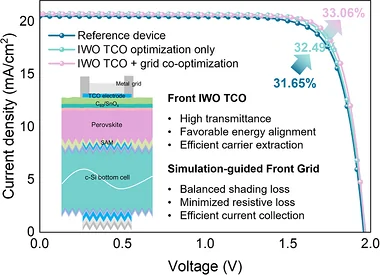

## 1 Introduction

Global cumulative photovoltaic (PV) capacity surged to nearly 3 TW in 2025; PV supplied over 10% of global electricity demand and approximately 12% of total power consumption, underpinning modern power systems as the world's fastest‐growing power generation technology [1]. Crystalline silicon solar cells, a pivotal component of photovoltaic installations, have achieved a record efficiency of 28.1% [2], which is extremely close to their theoretical efficiency limit of 29.4% [3]. To meet future energy demands, higher‐efficiency solar cells are urgently required, and perovskite/silicon tandem solar cells (PSTSCs) offer a promising solution to surpass the current bottleneck of single‐junction silicon solar cells [4, 5].

Over the past decade, researchers have conducted wide‐ranging studies on PSTSCs, including sub‐cell compatibility [6, 7, 8, 9, 10], functional layer materials [11, 12, 13, 14, 15], perovskite passivation [16, 17, 18, 19], and interconnected layer regulation [20, 21, 22, 23, 24]. Unfortunately, the front electrode of PSTSCs has not received sufficient attention, which may constitute one of the key factors limiting efficiency improvements. In currently known PSTSC configurations, the front electrode consists of a transparent conductive oxide (TCO) layer serving as the transparent electrode and an opaque metal grid electrode. The TCO is responsible for extracting carriers from the carrier transport layer (CTL) and delivering them to the metal electrodes via lateral transport, while the metal electrodes collect the current and feed it into the external circuit. In terms of TCOs, zinc‐doped indium oxide (IZO) has become the typical choice due to its favorable conductivity and light transmittance in the near‐infrared (NIR) region [23, 25]. However, most previous studies merely characterized the photovoltaic performance of IZO‐based devices without quantifying how work function (WF) and band offsets alter interfacial carrier dynamics [26]. Meanwhile, existing grid optimization largely relies on repeated device fabrication instead of predictive numerical simulation, leading to high experimental cost and inefficient parameter screening. For metal grids, a balance must be struck between shading losses and resistive losses in pursuit of optimal power output. According to Ebong et al. [27], excessively wide grid lines in silicon solar cells induce significant shading losses, which severely reduce the short‐circuit current density (*J_SC_*); conversely, insufficient width elevates series resistance (*R_S_*), thereby degrading the fill factor (FF). Siraj and co‐workers further investigated the influence of front grid metallization patterns on the performance of back surface field (BSF) silicon solar cells [28], achieving performance optimization by tuning the number of metal fingers and front contact resistance values. Consequently, well‐engineered front electrodes are essential for PSTSCs to fully unlock their power output potential.

In view of these gaps, this work integrates TCO band engineering and simulation‐guided metallization design to minimize overall front‐electrode power loss. Adopting W‐doped indium oxide (IWO) as the transparent electrode, which exhibits a more favorable WF and interfacial band alignment than IZO, facilitates efficient electron extraction and boosts device performance. Furthermore, our self‐developed simulation software was employed to investigate the impacts of metal‐electrode geometric parameters on output power, revealing that a balance between shading loss and resistive loss can be realized by regulating grid width and spacing. Subsequent experimental optimization identified the optimal metallization geometry with a 51.09 µm grid width and 2.5 mm spacing. Finally, the optimized PSTSCs achieved a power conversion efficiency (PCE) exceeding 33% with both high *J_SC_* and FF. This work provides practical guidelines for electrode design and metallization engineering in the industrialization of PSTSCs.

## 2 Results and Discussion

IZO and IWO films with a thickness of approximately 40 nm were deposited on quartz glass via reactive plasma deposition (RPD) to characterize the optoelectronic performance of these two types of TCO layers. As presented in Figure S1, the average transmittance of IZO and IWO reached 80.0% and 80.5% separately. This result confirmed that the RPD‐deposited IWO film maintained favorable optical transmittance. Specifically, IWO exhibited slightly lower transmittance than IZO in the 300–450 nm range, yet featured improved light transmittance for wavelengths above 450 nm, especially in the near‐infrared region. Hall effect measurements were performed to quantify the electrical properties of IZO and IWO films with a uniform thickness. As shown in Table S1, compared with IZO, the IWO film exhibited an increased carrier concentration from 3.55 × 10^20^ cm^−3^ to 3.88 × 10^20^ cm^−3^, accompanied by a slight improvement in carrier mobility. Thanks to its optimized carrier transport properties, IWO achieved a reduced sheet resistance from 99.0 to 88.5 Ω /sq, which effectively mitigated lateral resistive losses in the front transparent electrode.

We used Kelvin probe force microscopy (KPFM) to characterize the surface potential (SP) distribution of ALD‐deposited tin oxide (SnO_x_) films and two types of TCO films deposited by reactive plasma deposition (RPD) on top of them, as shown in Figure 1a–c. Compared with SnO_X_ films, IZO films showed slightly lower SP values, whereas IWO films displayed a substantial reduction. This indicated that IWO possessed a lower WF, favoring electron transport [29]. Furthermore, atomic force microscopy (AFM) characterization revealed that the root‐mean‐square (RMS) roughness decreased from 45.0 nm for ALD‐grown pristine SnO_x_ to 22.4 nm for the IZO and 25.8 nm for the IWO (Figure S2). The considerable reduction in surface roughness was mainly attributed to the planarization effect of RPD TCO films [30], which improved interfacial contact with the metal electrodes.

**FIGURE 1:**
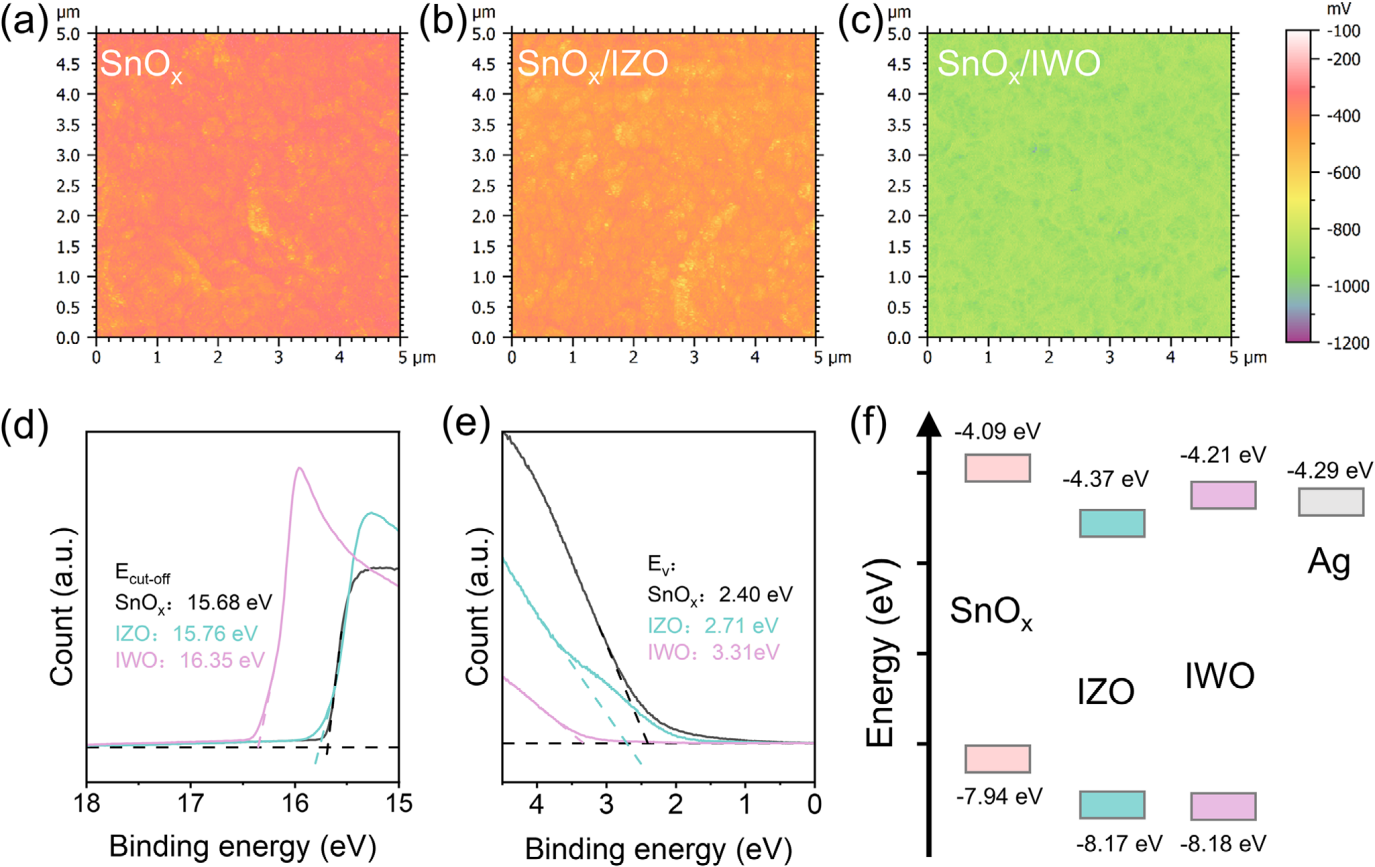
(a–c) KPFM images of SnO_x_, SnO_x_/IZO and SnO_x_/IWO films; (d–e) UPS spectra of SnO_x_, IZO and IWO films; (f) The energy level alignment schematics of front electrodes in tandem devices.

Ultraviolet photoelectron spectroscopy (UPS) was employed to characterize the band structures of the films. As presented in Figure 1d,e, the secondary electron cutoff edge of IWO shifted toward higher energy relative to SnO_x_ and IZO, corresponding to its lower WF of 4.87 eV. By contrast, the WFs of SnO_x_ and IZO were 5.54 and 5.46 eV, respectively, and the variation was consistent with KPFM characterizations. The valence band edge energies of SnO_x_, IZO, and IWO were determined to be 2.40, 2.71, and 3.31 eV, respectively. Figure 1f illustrated the energy level alignment of IZO and IWO transparent electrodes paired with the SnO_x_ buffer layer and Ag electrode. The diagram revealed that replacing IZO with IWO narrowed the energy offset between the electrode and adjacent functional layers [31].

We conducted steady‐state photoluminescence (PL) and time‐resolved photoluminescence (TRPL) tests to further investigate carrier dynamic processes. As depicted in Figure 2a, perovskite films integrated with an electron transport layer (ETL) and the IWO transparent electrode exhibited weaker PL emission intensity compared with those paired with IZO. The lower PL emission intensity for the IWO sample demonstrates its superior carrier extraction capability [32], originating from the favorable energy level alignment between SnO_x_ and IWO. Consistently, the shorter carrier lifetime observed for the IWO‐based sample in Figure 2b verified the accelerated electron collection within the perovskite/C_60_/SnO_x_/IWO structure [11]. PL intensity mappings revealed that perovskite stacks with an IWO transparent electrode delivered more homogeneous luminescence relative to IZO (Figure 2c,d), which was ascribed to the uniform SP distribution of the IWO film. Moreover, the average PL mapping intensity of the IWO‐based sample was lower than that of the IZO device, originating from suppressed radiative recombination owing to accelerated carrier extraction.

**FIGURE 2:**
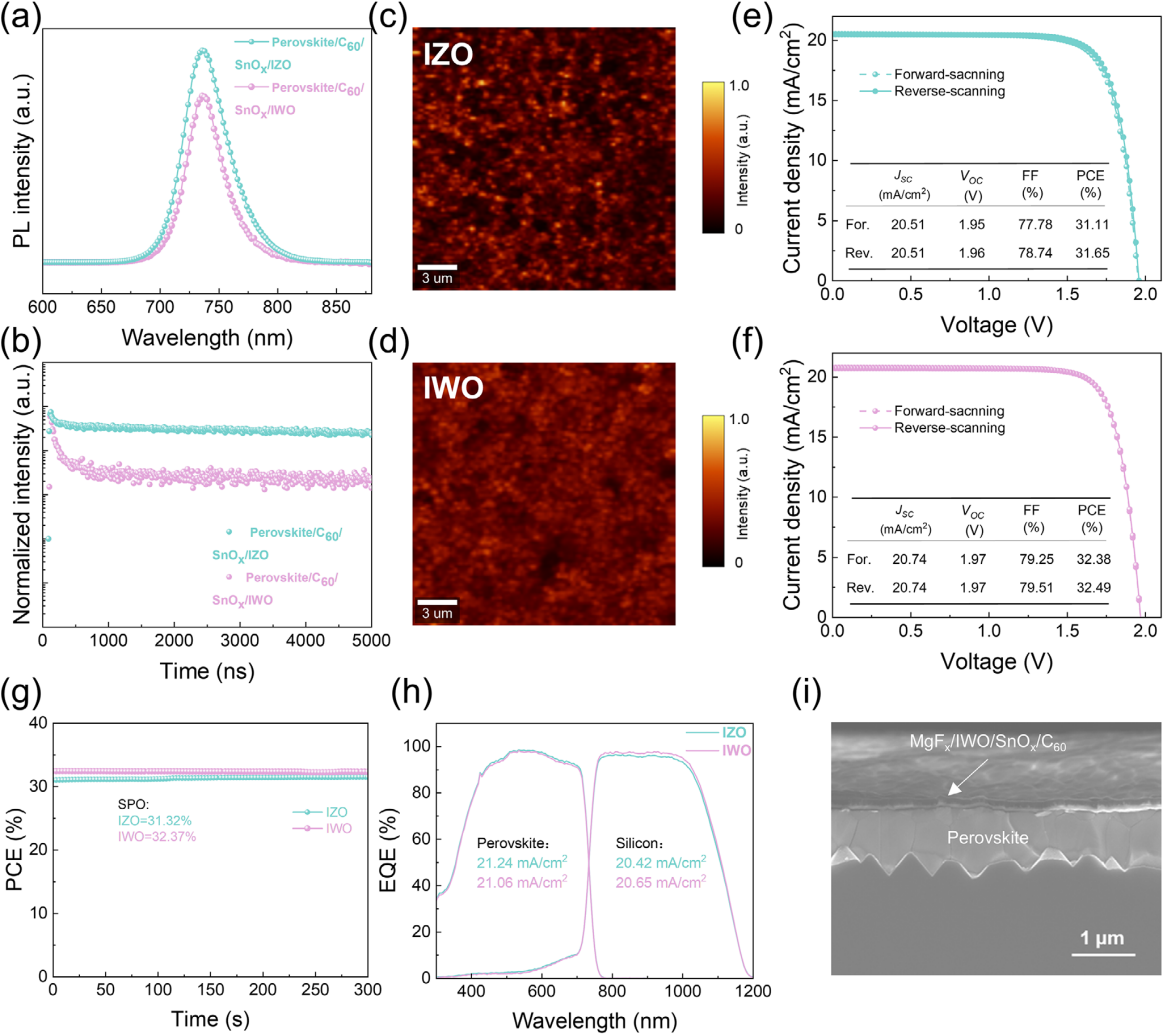
(a, b) PL and time‐resolved PL spectra of perovskite/C_60_/SnO_x_/TCO samples; (c, d) PL mappings of perovskite/C_60_/SnO_x_/TCO films; (e, f) *J–V* curves of the champion PSTSCs fabricated with IZO and IWO transparent electrodes, respectively; (g) Steady‐state power output of tandem devices with distinct TCO layers; (h) EQE spectra of IZO‐ and IWO‐based PSTSCs; (i) Cross‐sectional SEM micrograph of the IWO‐integrated tandem cell.

To clarify how the transparent electrode affects device performance, we fabricated two sets of PSTSCs with identical metal electrodes, employing IZO and IWO as the front transparent electrodes, respectively, and Figure S3a displayed a schematic illustration of the tandem device architecture. Figure S4 presented the statistical box plots of photovoltaic parameters for ten PSTSCs employing different transparent electrodes. Devices with IWO front electrodes delivered higher *J_SC_*, FF, and PCE relative to their IZO counterparts. Substitution of IZO with IWO raised both the median value and overall distribution range of *J_SC_*, which can be attributed to the enhanced near‐infrared transmittance of the IWO thin film. Benefiting from the optimized interfacial energy‐level alignment, the IWO group exhibited a marked improvement in FF. Collectively, these gains lead to a substantial enhancement in the PCE of IWO‐based PSTSCs. The forward and reverse scanning *J–V* curves of the champion tandem devices were displayed in Figure 2e,f. The IZO‐based tandem device achieved a maximum PCE of 31.65%, with a *J_SC_* of 20.51 mA/cm^2^, a *V_OC_* of 1.96 V, and an FF of 78.74%, and retained a steady‐state power output (SPO) efficiency of 31.32% over 300 s (Figure 2g). Benefiting from accelerated electron transport capability, the PSTSCs employing IWO transparent electrodes achieved a champion PCE of 32.49%, which stemmed from the FF elevation from 78.74% to 79.51%. Meanwhile, the *V_OC_* stayed steady at 1.97 V, while the *J_SC_* rose slightly to 20.74 mA/cm^2^, enabling the device to reach a SPO efficiency of 32.37%. In addition, negligible hysteresis was detected for both devices, verifying a well‐passivated perovskite.

The integrated current densities calculated from external quantum efficiency (EQE) spectra were 21.24 mA/cm^2^ for the perovskite top sub‐cell and 20.42 mA/cm^2^ for the silicon bottom sub‐cell in devices with an IZO electrode (Figure 2h). By contrast, the IWO‐based device yielded integrated currents of 21.06 mA/cm^2^ for the perovskite top cell and 20.65 mA/cm^2^ for the silicon bottom cell, which alleviated the current mismatch between the two sub‐cells. The EQE characterization further indicated that the PSTSC equipped with the IWO transparent electrode exhibited improved photo‐response in the near‐infrared region. This improvement originated from the higher transmittance of IWO thin films at near‐infrared wavelengths [33, 34], which accordingly raised the *J_SC_* of the tandem device. In addition, Figure 2i and Figure S3b,c displayed cross‐sectional SEM micrographs of the tandem devices, which clearly verified intimate interfacial contact among each functional layer.

To evaluate the long‐term reliability of the transparent electrodes, comparative stability tests were performed on IZO and IWO tandem devices. As shown in Figure S5, after 500 h of storage under ambient conditions (ISOS‐D‐1), the IZO‐based devices degraded to 94% of their initial PCE, whereas the IWO counterparts retained 95.4%. Figure S6 depicts the efficiency evolution of unencapsulated devices from both groups during 500 h of maximum power point tracking (MPPT) under 1‐sun illumination (ISOS‐L‐1). Under these more demanding operating conditions, the IZO sample retained merely 86.1% of its initial PCE, while the IWO device maintained 91.3%. These results indicate that replacing IZO with IWO does not compromise ambient storage stability and notably improves the operational lifetime of tandem devices under prolonged illumination.

Although a novel IWO was adopted as the transparent electrode, the performance of the tandem cell (especially its FF) still fell well below the theoretical limit. This discrepancy partially originates from series resistance induced by the non‐optimized metal grid design [35]. We developed a homemade simulation program to predict power losses originating from metal grid shading and resistive losses (Figures S7 and S8), which provided effective guidance for further device optimization. The detailed model establishment and software implementation procedures are presented in the Supplementary Information. The simulation incorporated multiple key physical parameters, including TCO sheet resistance (ρ_sq_), metal finger width (*W*) and height (*H*), grid spacing (*S*), metal finger resistance (ρ_m_), and TCO‐metal contact resistance (ρ_contact_). Among these variables, ρ_sq_, ρ_m_, ρ_contact,_ and metal finger height exhibit monotonic correlations with total power loss. In contrast, finger width and grid spacing present non‐monotonic trends owing to the inherent trade‐off between shading and resistive losses. Wider fingers with reduced spacing raise optical shading losses, which then become the dominant loss channel, whereas narrow fingers with larger spacing amplify resistive contributions, including TCO lateral transport and grid line resistance. Accordingly, grid geometry requires careful balancing of finger width and spacing to minimize overall front‐electrode power loss and maximize the device PCE. It is worth noting that this unit‐cell model adopts simplifying assumptions such as uniform photogenerated current and exclusive consideration of front‐electrode losses; thus, it provides fast qualitative guidance for grid optimization, and notable deviations from experimental results may occur for devices with severe inhomogeneity or local shunts.

To visualize the trade‐off between grid line geometric parameters and total power loss, we generated contour plots of combined power loss contributions from shading and resistive components (Figure 3a), with grid line *W* and *S* as variables; the simulation parameters were shown in Table S2. The color bar and contour lines quantitatively illustrated the magnitude of total power loss and divided the parameter space into two distinct regions. In the high‐loss region, increasing grid width and decreasing grid spacing sharply raised the total power loss ratio from 0.2 to 0.7, where shading loss dominated the overall power consumption. In contrast, the low‐loss region featured a well‐balanced contribution between shading loss and resistive loss, with the total loss ratio (*n*) consistently maintained below 0.1. The minimum power loss occurred under the conditions of *W* < 100 µm and *S* < 5 mm, which defines the optimal geometric parameter range for device fabrication. We further investigated the dependence of the *n*‐*W*‐*S* power loss relationship on ρ_sq_, as visualized in Figure 3b,c. The simulation results revealed that an increase in ρ_sq_ necessitates a reduction in *S* to maintain minimal power loss. Higher ρ_sq_ aggravates lateral transport losses across the TCO layer, and shortening the grid spacing effectively reduces the charge transport distance, thereby alleviating such resistive power dissipation. Notably, regardless of the ρ_sq_ value, the minimum power loss was consistently achieved at a fixed grid width of approximately 25 µm, verifying that 25 µm serves as a universally optimal grid‐width parameter for PSTSCs fabrication.

**FIGURE 3:**
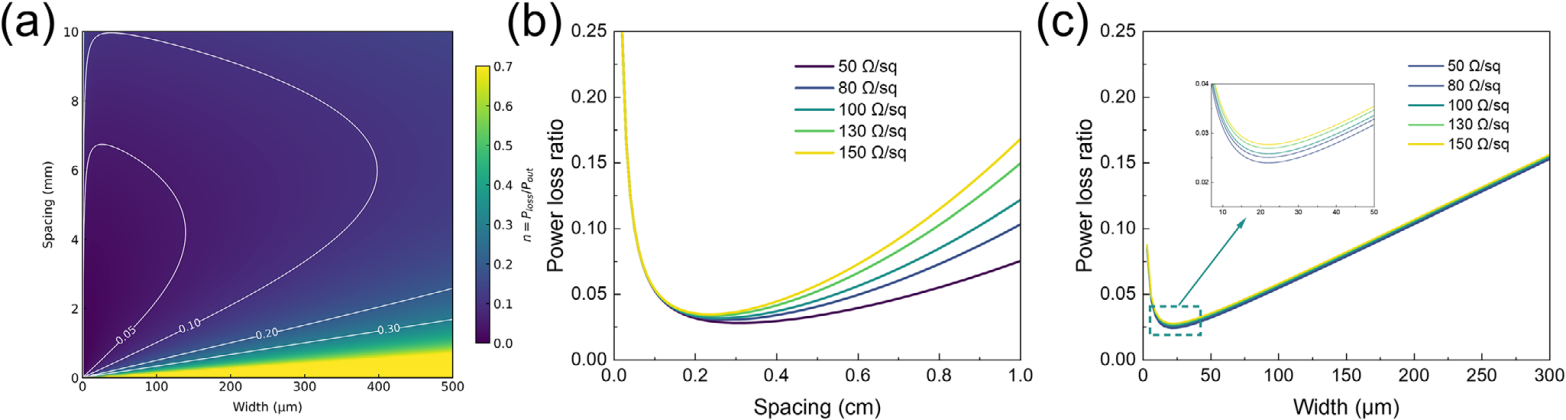
(a) Contour plot of the total power loss ratio as a function of metal grid width and spacing (the sheet resistance of the TCO was set to 100 Ω/sq); (b) and (c) Evolutions of the power loss ratio with spacing and width within the TCO sheet resistivity range of 50–150 Ω/sq. The inset in (c) magnifies the low‐loss region near the power loss minimum.

Considering the power losses originating from the width and spacing of metal grid lines that degrade device efficiency, we fabricated PSTSCs with a series of tailored grid geometric parameters to further optimize photovoltaic performance. We employed vapor deposition masks featuring distinct metal grid widths to fabricate a batch of tandem devices with variable grid line widths (Figure S9), while the grid spacing was fixed at 3.3 mm for all samples. Confocal microscopy measurements confirmed the as‐fabricated metal grid widths to be 51.09, 92.96, 130.65, and 176.71 µm (Figure 4a–d), respectively. The deviation between the fabricated grid width and the designed mask dimensions mainly stemmed from limited fabrication precision and inherent characteristics of the vapor deposition process. As the grid width increased from 50 to 170 µm, the theoretical shading ratio gradually climbed from 1.52% to 5.15% (Figure 4e), which directly suppressed photocurrent generation and therefore led to a continuous reduction in *J_SC_*. Figure 4f–i presented statistical distributions of photovoltaic parameters measured from four batches, with 10 devices per group. As the metal grid width rose, the *J_SC_* declined monotonically, which aligned well with the increased optical shading loss. In contrast, the *V_OC_* exhibited no obvious variation. Although the FF slightly improved with broader grid lines owing to diminished series resistance, the overall PCE dropped as grid width increased. Furthermore, no notable influence of grid width modification on the hysteresis index (HI) of devices was observed (Figure S10). Figure 4j presents the reverse‐scanning *J‐V* curves of the champion devices of each group. With the metal grid spacing fixed at 3.3 mm, the device fabricated with a grid width of 51.09 µm achieved the optimal photovoltaic performance, yielding a PCE of 32.57%, a *J_SC_* of 20.79 mA/cm^2^, a *V_OC_* of 1.968 V, and an FF of 79.61%. This trend arose because the detrimental effect of shading loss dominated over the performance benefits brought by lowered resistive loss. Although it was challenging to fabricate electrodes with ultra‐narrow metal grid widths via vapor deposition, further optimization of line width could alleviate adverse influences on the tandem devices. However, industrial metallization processes including screen printing [36, 37], electroplating [38, 39, 40] and laser transfer printing [41, 42] were capable of fabricating metal fingers with a width down to around 10 µm. Accordingly, the above findings still serve as a valuable reference for industrial metallization scheme design.

**FIGURE 4:**
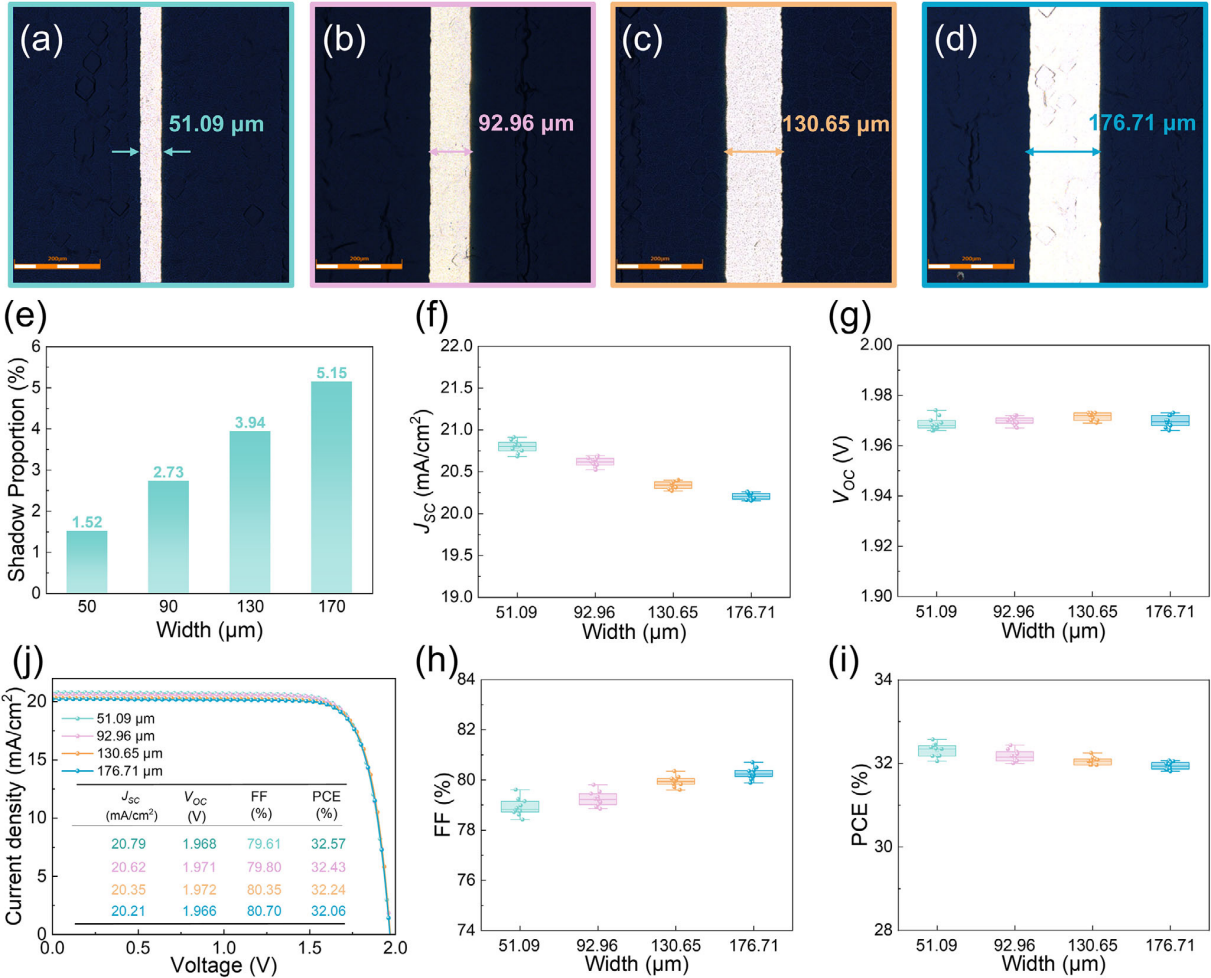
(a–d) Confocal microscopy images of metal grids with different widths; (e) Variation of the device shading ratio with increasing metal grid width; (f–i) Statistical box plots of photovoltaic parameters (*J_SC_*, *V_OC_*, FF, PCE) for 10 devices in each grid width group; (j) Reverse‐scanning *J–V* curves and photovoltaic parameters of the champion devices.

Subsequently, we fixed the metal grid width at approximately 50 µm and prepared PSTSCs with varied grid spacings via different deposition masks to explore how grid spacing influences device photovoltaic performance (Figure S11). As displayed in the digital photographs of the devices (Figure 5a–d), the corresponding metal grid spacings were 0.33, 0.25, 0.20, and 0.17 cm, respectively. As the metal grid spacing was reduced, the theoretical shading ratio rose from 1.52% to 2.99% (Figure 5e). Notably, the increase in optical shading induced by adjusting grid spacing was far less pronounced than that originating from variations in grid line width. In a similar trend, *J_SC_* dropped gradually as the grid spacing was narrowed (Figure 5f), while *V_OC_* exhibited negligible fluctuation (Figure 5g). As the metal grid spacing decreased from 0.33 to 0.25 cm, the FF was remarkably enhanced and kept rising with further narrowing of grid spacing (Figure 5h). This stemmed from the shortened lateral carrier transport distance induced by denser grid arrangement, which effectively cut down series resistance for charge collection [43]. Ultimately, the PCE peaked as the metal grid spacing was narrowed from 0.33 to 0.25 cm (Figure 5i), where optical shading loss and electrical resistive loss achieved an optimal balance. Likewise, variation in grid width showed no impact on device hysteresis (Figure S12), since the hysteresis effect of perovskite‐based solar cells mainly arises from ion migration, defect states, and interfacial charge accumulation. Figure 5j displayed the *J–V* curves and detailed parameters of the champion device from each group. The device with a grid spacing of 0.25 cm delivered the highest PCE of 33.06% (certified PCE of 32.28%, Figure S13), with a *J_SC_* of 20.72 mA/cm^2^, a *V_OC_* of 1.973 V, and an FF of 80.87%. This finding confirmed that moderately narrowing the metal grid spacing reduced series resistance and improved the FF without introducing excessive optical shading loss, hence optimizing the overall photovoltaic performance of PSTSCs.

**FIGURE 5:**
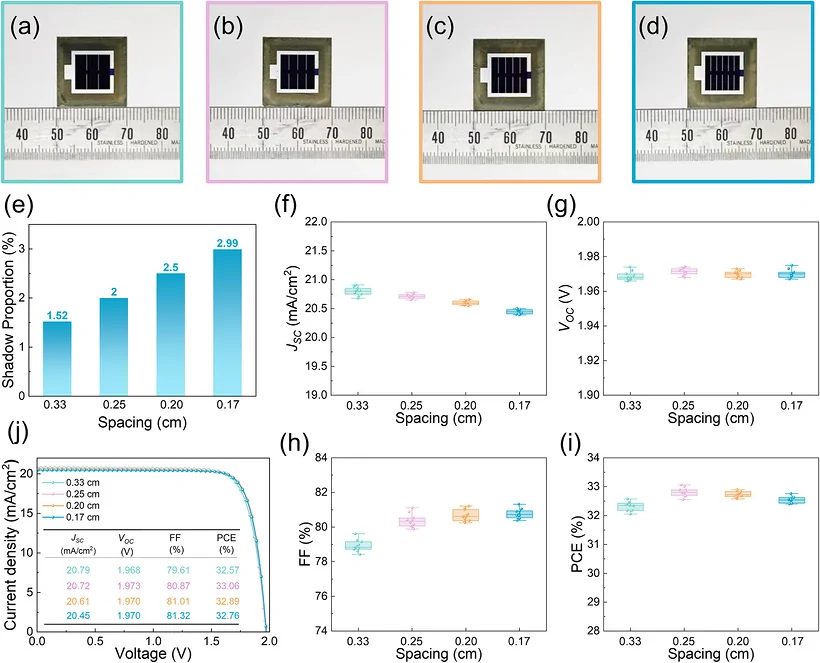
(a–d) Digital photographs of metal grids with different spacings; (e) Variation of the device shading ratio with decreasing metal grid spacings; (f–i) Statistical box plots of photovoltaic parameters (*J_SC_*, *V_OC_*, FF, PCE) for 10 devices in each grid spacing group; (j) Reverse‐scanning *J‐V* curves and photovoltaic parameters of the champion devices with distinct spacing.

## 3 Conclusion

In this work, high‐performance monolithic perovskite/silicon tandem solar cells were realized through the co‐optimization of TCO transparent layers and front metal grids. Replacing conventional IZO with low work function IWO optimized energy level alignment at the SnO_X_/TCO/Ag interface, reduced interfacial trap‐assisted recombination, and raised the FF from 78.74% to 79.51%. Benefiting from improved near‐infrared transmittance of IWO films, the *J_SC_* was slightly increased, lifting the baseline efficiency from 31.65% to 32.49%. To further reduce series resistive losses while avoiding photocurrent degradation, we utilized custom‐built simulation software to quantify power losses from front metal grids, investigated how grid geometry affects device performance, and verified that tuning grid width and spacing creates an optimal balance between shading and resistive losses. Guided by the simulation results, we fabricated tandem devices with gradient grid widths and spacings. Finally, we found that the device with a grid width of 50 µm and grid spacing of 2.5 mm attained a maximum PCE of 33.06% (certified PCE of 32.28%), with a *J_SC_* of 20.72 mA/cm^2^, a *V_OC_* of 1.973 V, and an FF of 80.87%. This work provides novel strategies and theoretical guidance for the performance optimization and industrialization of perovskite/silicon tandem solar cells.

## 4 Outlook

As PSTSCs scale toward large‐area commercialization, front‐electrodes play a decisive role in determining both the efficiency and cost of photovoltaic modules [44]. For TCO electrodes, we have only explored the performance of a single IWO material on small‐area devices; developing processing techniques to deposit large‐area, uniform TCO films within the thermal tolerance window of perovskite remains a critical research target. Meanwhile, reducing or eliminating the use of costly indium‐based materials represents an important route to cut down manufacturing costs. Furthermore, tuning the crystallization of TCO layers and employing stacked multi‐layer TCO configurations can deliver favorable optical responses and improved current matching for tandem devices while preserving low resistive losses.

Regarding metallization for tandem devices, although we have investigated the impacts of diverse grid parameters on device power loss in this work with particular emphasis on grid‐geometry design, different metallization routes bring additional practical factors into consideration [45]. Screen‐printing stands out as the most practically viable solution that can leverage existing silicon photovoltaic production lines. Constrained by the strict thermal budget of perovskite, low‐temperature curable silver pastes are highly demanded. Nevertheless, such pastes still suffer from high metal bulk resistivity and elevated TCO‐metal contact resistance. Copper‐based pastes or silver‐coated copper pastes can further lower metallization costs, yet the issue of copper oxidation must be properly addressed. Besides, inkjet printing and Flex‐Trail printing serve as alternative metallization approaches, whose practical feasibility for tandem devices has not yet been fully validated. Although electroplating enables excellent interfacial contact and low metal bulk resistivity, wet‐chemical solutions tend to corrode the perovskite absorber layer. Process compatibility thus becomes the major bottleneck, and integration challenges between wet processing and the perovskite/silicon tandem stack need to be resolved.

## Author Contributions

W. Liu, J. Liu, K. Yao, and Z. Liu directed and supervised the project. Y. Ji and J. Yu conceptualized and designed the experiments. Y. Ji, F. Wang, and P. Chen conducted device fabrication, optimization, and characterization. J. Li and Y. Luo conducted the simulation. L. Zhang and F. Meng aided in software development. Y. Ji, J. Yu, and W. Liu co‐wrote the manuscript. All authors have given approval to the final version of the manuscript.

## Conflicts of Interest

The authors declare no conflicts of interest.

## Supporting information




**Supporting File**: advs78191‐sup‐0001‐SuppMat.docx.

## Data Availability

The data that support the findings of this study are available from the corresponding author upon reasonable request. The simulation code for front‐grid optimization of perovskite/silicon tandem solar cells is publicly available at https://github.com/YuhuiJi/Front‐gird‐optimization‐for‐solar‐cells or can be obtained from the corresponding author.
