# Supplementary material for: Balancing Shading and Resistive Losses: 33% Efficient Perovskite/Silicon Tandem Solar Cells via Front‐Electrode Engineering
Source: Adv Sci (Weinh). 2026 Oct 6:e78191. Online ahead of print. doi: 10.1002/advs.78191 (PMC13643107; doi:10.1002/advs.78191)
Supplement: Supplementary file 1 [file ADVS-9999-e78191-s001.docx]

**Supplementary Information:**

**Balancing Shading and Resistance Losses: 33% Efficient Perovskite/Silicon Tandem Solar Cells via Front-Electrode Engineering**

Yuhui Ji^1, 2, †^, Fei Wang^2, †^, Junjun Li^2, †^, Yunren Luo^2,4^, Haichuan Zhang^6^, Qi Liu^2, 4^, Pengxu Chen^2, 4^, Kexin Yao^5^, Qiang Shi^2^, Fanying Meng^2, 4^, Liping Zhang^2, 4^, Chen Yang^3, 4^, Jiakai Liu^3, 4^, Zhengxin Liu^2, 4^, Wenzhu Liu^2,4, *^, and Jian Yu^1, *^

^1^ School of New Energy and Materials, Southwest Petroleum University, Chengdu 610500, China

^2^ Shanghai Institute of Microsystem and Information Technology, Chinese Academy of Sciences, Shanghai 201800, China

^3^ Xinjiang Technical Institute of Physics and Chemistry, Chinese Academy of Sciences, Urumqi 830011, China

^4^ University of Chinese Academy of Sciences (UCAS), Beijing 100049, China

^5^ School of Chemistry and Chemical Engineering, Institute of Advanced Inter-disciplinary Studies, Chongqing University, Chongqing 226532, China

^6^ Department of Mathematics, Shanghai Normal University, Shanghai 200234, China

^†^ These authors contributed equally to this work.

*Corresponding author: jianyu@swpu.edu.cn (J. Yu); wenzhu.Liu@mail.sim.ac.cn (W. Liu).

**Methods**

**Materials**

Materials used in this work include [4-(3,6-Dimethyl-9H-carbazol-9-yl)butyl]phosphonic Acid (Me-4PACz, 99%, TCI), cesium iodide (CsI, 99%, Xi’an Yuri Solar Co., Ltd.), methylammonium bromide (MABr, GreatCell), formamidinium iodide (FAI, GreatCell), lead(II) bromide (PbBr_2_, 99.8%, TCI), lead (II) iodide (PbI_2_, 99.9%, TCI), phenethylammonium bromide (PEABr, GreatCell), ethanediamine dihydroiodide (EDAI_2_, GreatCell), Fullerene (C_60_, 99%, Xi’an Yuri Solar Co., Ltd.), isopropanol (IPA, 99.5%, AcrosReal), N, N-dimethylformamide (DMF, 98.8%, AcrosReal), dimethylsulfoxide (DMSO, 99.7%, AcrosReal), chlorobenzene (CB, 99.8%, AcrosReal), zinc-doped indium oxide (IZO, 10 wt% ZnO, Changsha Enam Optoelectronic Material Co., Ltd.), W-doped indium oxide (IWO, 10 wt% W_2_O_3_, Changsha Enam Optoelectronic Material Co., Ltd.) and silver (Ag, 99.99%, Fuzhou Yingfeixun Optoelectronics Technology Co., Ltd.). All chemicals were used as received without further treatment.

**Fabrication of silicon bottom sub-cells**

The 300 μm n-type czochralski (CZ) silicon wafers were first conducted to remove the surface saw damage through an etching step and then textured by a relatively low concentrate aqueous solution of KOH to obtain a double-sided submicron pyramid structure and cleaned with a standard RCA and HF dip process. Hydrogenated intrinsic amorphous (i-a-Si:H) layers with a thickness of approximately 5 nm was deposited via plasma-enhanced chemical vapor deposition (PECVD) system with a temperature of 200 °C. Immediately, n-doped hydrogenated nanocrystalline silicon oxide (nc-SiO_X_:H) and p-doped nc-SiO_X_:H layers with thicknesses of about 15 nm and about 20 nm were deposited on the front and rear sides, respectively. Tungsten-doped indium oxide (IWO) layers were prepared by reactive plasma deposition (RPD) on both sides with a thickness of 20 nm as the interconnection layer and 40 nm as the rear contact, respectively. Ag was prepared on the back side by screen printing and cured at about 200 °C to ensure favorable current transfer and thermal evaporation deposited 150 nm MgF_X_ as a back reflector to increase NIR absorption. Finally, full-size solar cell units were segmented into 2 cm × 2 cm ready-to-use bottom cells using a laser scribing apparatus.

**Fabrication of perovskite/silicon tandem solar cells**

The perovskite top sub-cells with p-i-n configuration were fabricated on the silicon bottom cell. For hole transport layer (HTL), 1 mg/ml Me-4PACz in ethanol was used and spin-coated on the obtained silicon sub-cells at 5000 rpm for 30 s and annealed at 100 °C for 10min. A 1.7 M Cs_0.05_FA_0.8_MA_0.15_Pb(I_0.75_Br_0.25_)_3_ perovskite precursor solution was prepared by dissolving a mixture of FAI, MABr, CsI, PbI_2_, and PbBr_2_ in a mixed solvent of DMF and DMSO with a volume ratio of 4:1. The precursor solution was stirred for over 4 hours, and then filtered using a 0.22 μm PTFE membrane. For absorbing layer, wide band-gap perovskite films prepared on the above substrates using one-step anti-solvent spin-coating method and were annealed for 15 min at 100 °C. Then, the films were post-treated with EDAI_2_ +PEABr in IPA solution for passivation. Subsequently, 10 nm C_60_ and 15 nm SnO_2_ were sequentially prepared as electron transport layer (ETL) and buffer layer using thermal evaporation deposition and atomic layer deposition (ALD).

**Front Grid fabrication**

40 nm IWO or IZO was deposited under the PRD Power of 150 W on the SnO_2_ buffer layer at 100 °C to serve as a transparent electrode, with an Ar/O₂ flow ratio of 100/30. Silver grids were deposited via thermal evaporation using masks with distinct patterns. The grids spacing was fixed at 3.3 mm and deposited with widths of 50, 90, 130, and 170 μm, respectively. Additionally, we maintained a 50 μm grid width while varying the spacing from 3.3 to 1.5 mm. The deposition rate during the grid preparation process was uniformly kept at 1 Å/s, with a thickness of 500 nm. Finally, a 110 nm MgF_X_ anti-reflection layer was prepared in the thermal evaporation chamber.

**Characterization**

Surface morphology and electronic properties were investigated using atomic force microscopy (AFM) and Kelvin probe force microscopy (KPFM) on a Bruker Dimension Edge system. Ultraviolet photoelectron spectroscopy (UPS) was obtained using a Thermo Scientific ESCALab250Xi multifunctional photoelectron spectrometer. The excitation source for the UPS test was HeI, with an energy of 21.22 eV, and a bias voltage of -10 eV was applied during the test. Edinburgh FLSP1000 spectrophotometer with 440 nm picosecond pulsed diode laser excitation source was used to quantify static PL and TRPL of perovskite films. The *J-V* measurements in this study are based on the AAA-grade solar simulator manufactured by Yamashita Denso, with the equipment model number of YSS-T155-2M. The cells J-V tests in this work were conducted in the standard solar cell test conditions: the simulated irradiation of the sunlight was 100 mW/cm^2^, and the spectral distribution used was AM1.5, and the ambient temperature was maintained at 25 °C. And the active area defined by the aperture shade mask of tandem solar cells was 1.0 cm^2^, respectively. A QE-R equipment from Enli Technology Co. Ltd. was used to acquire the matching EQE spectrum of solar cells. Light emitting diodes with emission peaks at 850 and 400 nm were used as bias illumination to measure the top and bottom sub-cells. Scanning electron microscopy (SEM) images were obtained using Oxford equipment.

The microstructural features of the grids were examined using an Olympus LEXT OLS5100 3D laser confocal microscope. The confocal optical system employed a 405 nm laser diode light source and a high-sensitivity photomultiplier tube for acquiring confocal images.

**Computational methodology**

1. Computational Model Construction:

To establish a reliable experimental framework, we first constructed computational models to guide the design of grid line parameters. As illustrated in **Fig. S5(a)**, under device operating conditions, the generated photocurrent first undergoes lateral transport within the TCO layer until it reaches the metal grid lines. From this point, the current flows directionally through the grid lines, is subsequently collected by the collecting electrodes, and finally conducted to the external circuit. Building on previous publications^S1-3^ addressing transfer losses in solar cells, we systematically summarized the power loss components introduced by the front electrode system: these include shading losses, lateral transport losses in the TCO layer, contact losses at the TCO-metal electrode interface, and grid line resistance losses, as schematically presented in **Fig. S5(b)**.

To simplify the computational model while retaining critical physical insights, we defined a unit cell with a span of half the collecting electrodes spacing and half the grid line spacing, as shown in **Fig. S5(c)**. In this unit cell, L denotes the lateral current transport length along the grid line, S represents the grid line spacing, and W is the grid line width. By quantifying the power loss components within this unit cell, we can efficiently evaluate the impact of grid structural parameters on overall device output performance.

2. Power losses analysis:

2.1 The power loss caused by shadow

When light is incident on the surface of a perovskite/silicon tandem solar cell (PSTSC), the opaque metal grid lines block a portion of photons from reaching the active layer, resulting in reduced photocurrent and output power. This phenomenon is thus defined as shading loss, and we denote this specific power loss component as *P_shadow_*. And the power loss ratio is as follows:

$$\begin{aligned} \frac{P_{shadow}}{P_{out}}\approx\frac{{Area}_{shadow}\cdot J_{mp}\cdot V_{mp}}{{Area}_{total}\cdot J_{mp}\cdot V_{mp}}=\frac{{Area}_{shadow}}{{Area}_{total}}=\frac{W}{S}\#\left( 1 \right) \end{aligned}$$

Here, *J_mp_* and *V_mp_* represent the current density and voltage at the maximum power point operating condition, respectively.

2.2 The power loss from the sheet resistance of TCO

The sheet resistance of TCO films constitutes another critical source of power loss in PSTSCs. The sheet resistance of such films can be, to a certain extent, analogous to the emitter resistance of conventional silicon solar cells, providing a reference for subsequent loss calculations. We first assume the output current of a single PSTSC device is *I_0_*; the relationship between the current and its distance from the grid line is expressed by the following formula:

$$\begin{aligned} I\left( x \right)=I_{0}\frac{x}{\frac{s}{2}}\#\left( 2 \right) \end{aligned}$$

The power loss generated by the lateral transport of current in the TCO (*P_TCO_*) can be calculated using the following formula:

$$\begin{aligned} P_{TCO}=\int_{0}^{\frac{S}{2}} \frac{\rho_{sq}\cdot I\left( x \right)^{2}}{L}dx=\frac{\rho_{sq}\cdot S\cdot I_{0}^{2}}{6}\#\left( 3 \right) \end{aligned}$$

$$\begin{aligned} \frac{P_{TCO}}{P_{out}}=\frac{\rho_{sq}\cdot S\cdot I_{0}^{2}}{6}\cdot\frac{1}{I_{0}\cdot V_{mp}}=\frac{\rho_{sq}\cdot S\cdot I_{0}}{6\cdot V_{mp}}=\frac{\rho_{sq}\cdot S^{2}\cdot J_{mp}}{12\cdot V_{mp}}\#\left( 4 \right) \end{aligned}$$

Where *ρ_sq_* indicates the sheet resistivity of TCO.

2.3 The power loss due to contact resistance between TCO and metal electrodes

The contact interface between TCO and metal electrodes constitutes another critical source of power dissipation in PSTSCs. The power loss arising from this contact resistance (*P_contact_*) is primarily governed by two key factors: contact resistivity (*ρ_contact_*) and the effective contact area, and it can be quantified via the following formula:

$$\begin{aligned} P_{contact}=I_{0}^{2}\cdot\frac{\rho_{contact}}{L\cdot W}\#\left( 5 \right) \end{aligned}$$

$$\begin{aligned} \frac{P_{contact}}{P_{out}}=I_{0}^{2}\cdot\frac{\rho_{contact}}{L\cdot W}\cdot\frac{1}{V_{mp}\cdot I_{0}}=\frac{\rho_{contact}\cdot S\cdot J_{mp}}{W\cdot V_{mp}}\#\left( 6 \right) \end{aligned}$$

2.4 The power loss caused by the line resistance of the metal grids

As another pivotal power loss source in PSTSCs, the power dissipation induced by grid line resistance (*P_Rm_*) exerts a notable impact on the device’s output performance, which is primarily dependent on the grid lines' geometric parameters and intrinsic material properties. From a fundamental physics perspective, the relationship between current and position along the grid lines is described as follows:

$$\begin{aligned} I\left( x \right)=I_{0}\frac{x}{L}\#\left( 7 \right) \end{aligned}$$

The formula for power loss due to grid line resistance is as follows:

$$\begin{aligned} {dP}_{R_{m}}=I^{2}dR_{m}=I^{2}d\left( \frac{\rho_{m}\cdot x}{W\cdot H} \right)\#\left( 8 \right) \end{aligned}$$

$$\begin{aligned} P_{R_{m}}=\int_{0}^{L} \frac{I^{2}\left( x \right)\cdot\rho_{m}}{W\cdot H}dx=\frac{\rho_{m}\cdot L\cdot I_{0}^{2}}{3W\cdot H}\#\left( 9 \right) \end{aligned}$$

$$\begin{aligned} \frac{P_{R_{m}}}{P_{out}}=\frac{\rho_{m}\cdot L\cdot I_{0}^{2}}{3W\cdot H}\cdot\frac{1}{V_{mp}\cdot I_{0}}=\frac{\rho_{m}\cdot L^{2}\cdot S\cdot J_{mp}}{3W\cdot H\cdot V_{mp}}\#\left( 10 \right) \end{aligned}$$

Here, *ρ_m_* and *H* represent the resistivity and height of the metal grid lines, respectively.

Synthesizing the analyses of shading loss, TCO sheet resistance loss, TCO-metal contact resistance loss, and grid line resistance loss above, the equation for the power loss induced by the PSTSC's front electrode system accounting for the proportion of total output power—is given as follows:

$$\begin{aligned} n=\frac{P_{loss}}{P_{out}}=\frac{W}{S}+\frac{\rho_{sq}\cdot S^{2}\cdot J_{mp}}{12\cdot V_{mp}}+\frac{\rho_{contact}\cdot S\cdot J_{mp}}{W\cdot V_{mp}}+\frac{\rho_{m}\cdot L^{2}\cdot S\cdot J_{mp}}{3W\cdot H\cdot V_{mp}}\#\left( 11 \right) \end{aligned}$$

Based on the derived total power loss equation, we identified key dependencies of the front electrode power loss ratio (denoted as *n*) on device parameters: n is directly proportional to *J_mp_* and inversely proportional to *V_mp_*. This finding underscores the advantage of multi-junction solar cell technology in reducing *n*—transitioning from the "high current, low voltage" characteristic of single-junction silicon cells to the "high voltage, low current" profile of PSTSCs, with additional junctions promising even higher voltages and lower currents to further mitigate losses.

Furthermore, the material resistivity was found to exert a clear influence on *n*: lower resistivity consistently reduces *n*, though this must be balanced against practical constraints such as economic feasibility and manufacturing complexity. Notably, the current transport distance and grid line height also significantly regulate n: a larger *L* correlates with higher *n*, which explains why small-area lab-scale devices typically outperform commercial large-area ones—since increasing device area inevitably extends *L*. Conversely, increasing *H* reduces the resistance of current transport along grid lines, thereby lowering *n*; however, this approach also introduces economic and technical challenges in scalable fabrication.

The key geometric parameters of grid lines (specifically *W* and *S*) exhibit a mutually restrictive relationship that critically regulates the front electrode power loss ratio. It is noteworthy that a larger *W* combined with a smaller *S* directly drives a higher *n*, where shading loss becomes the dominant contributing factor. Conversely, a smaller *W* paired with a larger *S* also elevates *n*, but in this case, resistive losses (encompassing TCO transport loss, TCO-metal contact loss, and grid line resistance loss) take precedence. This inherent trade-off clearly indicates that grid line geometric design must strike a delicate balance between width and spacing, thereby minimizing total power loss and ultimately achieving maximum PCE.

Based on the power loss calculation framework established above, we conducted systematic simulations using a Python program. To further facilitate engineers in obtaining optimal grid line designs for maximizing device performance, we have developed a dedicated optimization software. **Fig. S6** shows the graphical user interface (GUI) of our self-developed grid optimization simulation software, which contains four functional parts. Part 1: inputs standardized device electrical and structural parameters. Part 2: sets the value ranges of grid width W and spacing S to cut computation time. The Start Grid Search button in Part 3 triggers the optimization algorithm. Part 4: displays a power-loss contour plot with a red dot marking the optimal W and S. A pop-up window simultaneously outputs the exact optimized parameter values.

The complete code of our custom-built simulation program is presented below for reference.

```
import numpy as np
from PySide6.QtWidgets import (
    QApplication, QWidget, QGridLayout, QLabel, QLineEdit, QPushButton,
    QDoubleSpinBox, QCheckBox, QFileDialog, QMessageBox
)
from PySide6.QtWidgets import QGroupBox, QHBoxLayout, QVBoxLayout
from PySide6.QtCore import Qt
import matplotlib
import matplotlib.font_manager as fm
matplotlib.use("QtAgg")
matplotlib.rcParams['font.family'] = 'Microsoft YaHei'
matplotlib.rcParams['axes.unicode_minus'] = False
from matplotlib.backends.backend_qtagg import FigureCanvasQTAgg
from matplotlib.figure import Figure

def compute_n(W, S, params):
    """
    n = P_loss / P_out
    n = W/S + (ρ_sq · S² · J_mp) / (12 · V_mp)
        + (ρ_contact · S · J_mp) / (W · V_mp)
        + (ρ_m · L² · S · J_mp) / (3 · W · H · V_mp)

    W: grids' width (μm)
    S: grids' spacing (mm)
    H: grids' height (μm)
    L: Transfer distance (cm)
    J_mp: current density at maximum power point (mA/cm²)
    V_mp: voltage at maximum power point (mV)
    ρ_sq: sheet resistivity of TCO (Ω/sq)
    ρ_contact: contact resistivity between TCO and metal grids (mΩ·cm²)
    ρ_m: metal grids line resistivity (μΩ·cm)
    """
    H = params["H"]
    L = params["L"]
    J_mp = params["J_mp"]
    V_mp = params["V_mp"]
    rho_sq = params["rho_sq"]
    rho_contact = params["rho_contact"]
    rho_m = params["rho_m"]
    term1 = (W / 1000) / S

    S_cm = S / 10
    term2 = (rho_sq * S_cm * S_cm * J_mp) / (12 * V_mp)

    W_cm = W / 10000
    term3 = (rho_contact * S_cm * J_mp) / (W_cm * V_mp)

    H_cm = H / 10000
    term4 = (rho_m * 1e-6 * L * L * S_cm * J_mp) / (3 * W_cm * H_cm * V_mp)

    n = term1 + term2 + term3 + term4
    return n

class MplCanvas(FigureCanvasQTAgg):
    def __init__(self):
        fig = Figure(figsize=(5, 4))
        self.ax = fig.add_subplot(111)
        super().__init__(fig)

class GridSearchApp(QWidget):
    def __init__(self):
        super().__init__()
        self.setWindowTitle("Grid Optimization (Grid Search)")
        self.setMinimumSize(1000, 850)

        self.colorbar = None

        layout = QGridLayout(self)

        param_box = QGroupBox("Input Parameters")
        param_layout = QGridLayout()

        self.inputs = {}

        labels = [
            ("H — grids' height (μm)", "H"),
            ("L — Transfer distance (cm)", "L"),
            ("J_mp — current density at maximum power point (mA/cm²)", "J_mp"),
            ("V_mp — voltage at maximum power point (mV)", "V_mp"),
            ("ρ_sq — sheet resistivity of TCO (Ω/sq)", "rho_sq"),
            ("ρ_contact — contact resistivity between TCO and metal grids (mΩ·cm²)", "rho_contact"),
            ("ρ_m — metal grids line resistivity (μΩ·cm)", "rho_m"),
        ]

        for i, (label_text, key) in enumerate(labels):
            lbl = QLabel(label_text)
            inp = QDoubleSpinBox()
            inp.setRange(0.0, 1e9)
            inp.setDecimals(6)
            inp.setValue(1.0)
            self.inputs[key] = inp
            param_layout.addWidget(lbl, i, 0)
            param_layout.addWidget(inp, i, 1)

        param_box.setLayout(param_layout)
        layout.addWidget(param_box, 0, 0, 1, 2)

        ws_box = QGroupBox("Optimization Parameters: W — grids' width (μm), S — grids' spacing (mm)")
        ws_layout = QGridLayout()

        self.w_min = QDoubleSpinBox(); self.w_min.setRange(0, 1e6); self.w_min.setValue(1)
        self.w_max = QDoubleSpinBox(); self.w_max.setRange(0, 1e6); self.w_max.setValue(100)
        self.s_min = QDoubleSpinBox(); self.s_min.setRange(0, 1e6); self.s_min.setValue(0.1)
        self.s_max = QDoubleSpinBox(); self.s_max.setRange(0, 1e6); self.s_max.setValue(10)

        self.w_unlimit = QCheckBox("W unlimited (positive range)")
        self.s_unlimit = QCheckBox("S unlimited (positive range)")

        ws_layout.addWidget(QLabel("W min (μm):"), 0, 0)
        ws_layout.addWidget(self.w_min, 0, 1)
        ws_layout.addWidget(QLabel("W max (μm):"), 0, 2)
        ws_layout.addWidget(self.w_max, 0, 3)

        ws_layout.addWidget(self.w_unlimit, 1, 0, 1, 4)

        ws_layout.addWidget(QLabel("S min (mm):"), 2, 0)
        ws_layout.addWidget(self.s_min, 2, 1)
        ws_layout.addWidget(QLabel("S max (mm):"), 2, 2)
        ws_layout.addWidget(self.s_max, 2, 3)

        ws_layout.addWidget(self.s_unlimit, 3, 0, 1, 4)

        ws_box.setLayout(ws_layout)
        layout.addWidget(ws_box, 1, 0, 1, 2)

        self.run_btn = QPushButton("Start Grid Search")
        self.run_btn.clicked.connect(self.run_search)
        layout.addWidget(self.run_btn, 2, 0, 1, 2)

        self.canvas = MplCanvas()
        layout.addWidget(self.canvas, 3, 0, 1, 2)

    def run_search(self):
        params = {k: inp.value() for k, inp in self.inputs.items()}

        Wmin = 0.01 if self.w_unlimit.isChecked() else self.w_min.value()
        Wmax = 1e6 if self.w_unlimit.isChecked() else self.w_max.value()

        Smin = 0.01 if self.s_unlimit.isChecked() else self.s_min.value()
        Smax = 1e6 if self.s_unlimit.isChecked() else self.s_max.value()

        Ws = np.linspace(Wmin, Wmax, 80)
        Ss = np.linspace(Smin, Smax, 80)

        W_grid, S_grid = np.meshgrid(Ws, Ss)

        n_values = np.zeros_like(W_grid)

        for i in range(len(Ss)):
            for j in range(len(Ws)):
                n_values[i, j] = compute_n(W_grid[i, j], S_grid[i, j], params)

        idx = np.unravel_index(np.argmin(n_values), n_values.shape)
        best_W = W_grid[idx]
        best_S = S_grid[idx]
        best_n = n_values[idx]

        QMessageBox.information(
            self, "Optimal Result",
            f"Minimum n = {best_n:.6f}\nOptimal W = {best_W:.6f} μm\nOptimal S = {best_S:.6f} mm"
        )

        if self.colorbar is not None:
            self.colorbar.remove()
            self.colorbar = None

        self.canvas.ax.clear()
        contour = self.canvas.ax.contourf(W_grid, S_grid, n_values, levels=40, cmap="viridis")
        self.canvas.ax.scatter([best_W]
, [best_S], color="red", s=60, label="Optimal point")
        self.canvas.ax.set_xlabel("W (μm)")
        self.canvas.ax.set_ylabel("S (mm)")
        self.canvas.ax.set_title("n minimization — Grid Search Result")
        self.canvas.ax.legend()
        self.colorbar = self.canvas.figure.colorbar(contour)
        self.canvas.figure.tight_layout()
        self.canvas.draw()

if __name__ == "__main__":
    app = QApplication([])

    win = GridSearchApp()
    win.show()

    app.exec()
```

**Fig. S1.** Optical transmittance of IZO and IWO film.

**Fig. S2.** AFM images of SnO_X_, SnO_X_/IZO and SnO_X_/IWO films. AFM results revealed that the pristine SnO_X_ film possessed an RMS roughness of 45.0 nm. Covering SnO_X_ with IZO and IWO reduced the surface RMS roughness to 22.4 nm and 25.8 nm respectively.

**Fig. S3.** (a), the schematic illustration of the perovskite/silicon tandem device in this work; (b and c), SEM cross-sectional image of the rear side of the device and its corresponding magnified detail view. Fig. S2(b) presents a low-magnification cross-sectional SEM micrograph of the screen-printed rear Ag electrode, with the measured thickness of the silver varying from roughly 4.1 μm to 10.8 μm. Fig. S2(c) further reveals tight bonding among silver paste particles and favorable interfacial contact between the printed Ag layer and the rear-side TCO film.

**Fig. S4.** Box plots showing the statistical distribution of photovoltaic performance parameters for 10 independently fabricated tandem devices in each group equipped with different TCO transparent electrodes. The median values and overall distribution ranges of *J_SC_* were elevated after replacing IZO with IWO, which originated from the improved near-infrared light transmittance of IWO films. Benefiting from the optimized energy level alignment, FF showed a notable upward shift in the IWO group. As a comprehensive result, the PCE of the IWO devices was significantly promoted.

**Fig. S5.** Stability of unencapsulated devices exposed to RH=40% at atmosphere.

**Fig. S6.** The MPP tracking of unencapsulated tandem devices under 1-sun illumination.

**Fig. S7.** (a), Schematic illustration of the current transport path in PSTSCs; (b), Schematic diagram of four key power loss components induced by the front electrode system; (c), Schematic of the unit cell used for simplified calculations, where L denotes the lateral current transport length along the grid line, S is the grid line spacing, W is the grid line width.

**Fig. S8.** Graphical user interface of the custom-built simulation software for front metal grid optimization. Part 1: inputs standardized device electrical and structural parameters. Part 2: sets the value ranges of grid width W and spacing S to cut computation time. The Start Grid Search button in Part 3 triggers the optimization algorithm. Part 4: displays a power-loss contour plot with a red dot marking the optimal W and S.

**Fig. S9.** Evaporation masks for metal grids with different preset widths, with white areas defining silver electrodes. Owing to mask fabrication precision limit and vapor deposition effects, the real width is usually larger than the designed value, so all labeled widths in the figure are measured practical values.

**Fig. S10.** Statistical distribution of hysteresis index (HI) for tandem devices with varied grid widths

**Fig. S11.** Photomask for thermal evaporation of metal grids with diverse grid finger spacings, with a fixed designed finger width of approximately 50 μm. The white regions denote the patterned silver electrode areas.

**Fig. S12.** Statistical distribution of hysteresis index (HI) for tandem devices with varied grid spacings.

**Fig. S13.** J-V characteristics certification report of the perovskite/silicon tandem solar cell.

**Table S1**. Electrical properties of IZO and IWO films: n stands for carrier concentration, μ for carrier mobility, and Rsq for sheet resistance.

| Film | Thickness (nm) | n (×10^20^ cm^-3^) | μ (cm^2^/V s) | Rsq (Ω/sq) |
| --- | --- | --- | --- | --- |
| IZO | 40 nm | 3.53 | 44.6 | 99 |
| IWO | 40 nm | 3.89 | 45.4 | 88.5 |

**Table S2**. Parameters adopted for simulating power loss induced by front-electrode metal grids

| Parameters | Values | Units |
| --- | --- | --- |
| J_mp_ | 19.5 | mA/cm^2^ |
| V_mp_ | 1750 | mV |
| L | 1 | cm |
| H | 500 | nm |
| ρ_sq_ | 50-150 | Ω/sq |
| ρ_m_ | 1.6 | μΩ∙cm |
| ρ_contact_ | 0.3 | mΩ∙cm^2^ |

**Supplementary References**

(S1) Ebong, A.; Chen, N. Metallization of crystalline silicon solar cells: A review. In *High Capacity Optical Networks and Emerging/Enabling Technologies*, 12-14 Dec. 2012, 2012; pp 102-109.

(S2) Hu, C.; Li, K.; Li, X.; Li, J.; Sun, F.; Fan, X.; Yang, T.; Wang, R.; Deng, A. Optimizing front grid electrodes of flexible CIGS thin film solar cells with different shapes. *Solar Energy* **2024**, *284*.

(S3) Rehman, A. u.; Van Kerschaver, E. P.; Aydin, E.; Raja, W.; Allen, T. G.; De Wolf, S. Electrode metallization for scaled perovskite/silicon tandem solar cells: Challenges and opportunities. *Progress in Photovoltaics: Research and Applications* **2021**, *31* (4), 429-442.
